# Supplementary material for: Comprehensive assessment of physiological responses in women during the ESA dry immersion VIVALDI microgravity simulation
Source: Nat Commun. 2023 Oct 9;14:6311. doi: 10.1038/s41467-023-41990-4 (PMC10562467; doi:10.1038/s41467-023-41990-4)
Supplement: Supplementary file 1 — Supplementary Information [file 41467_2023_41990_MOESM1_ESM.pdf]

Comprehensive assessment of physiological responses in women during the ESA dry immersion VIVALDI microgravity simulation

Supplementary Information

Table.1 | Baseline general characteristics
Baseline characteristics (mean±SEM) at B3 (B2 for $\dot{V}O_2$max, B4 for basal resting metabolic rate, RMR). Height was assessed in standing position. Morning heart rate (HR) and blood pressure (SBP, DBP) were measured around 07:00 by a brachial pneumatic cuff device.

**Age** (year)	**Height** (cm)	**Weight** (kg)	**BMI** (kg/m2)	**$\dot{V}O_2$ max** (mL.kg^{-1}.min^{-1})	**RMR** (kcal/day)	**Morning HR** (bpm)	**Morning SBP** (mmHg)	**Morning DBP** (mmHg)
29±1	164.8±1.4	59.3±1.5	21.8±0.4	38.7±1.5	1334±25	64±2	109±3	64±1

Supplementary Table.2 | Blood assessment performed by MEDES at B4 and R1 for medical safety reasons
Data are mean ± s.e.m.

	**Normal ranges**	**B4**	**R1**
**RBC** - red blood cells (10^{12}.L^{-1})	[3.8 - 5.9]	4.28±0.06	4.18±0.06
**Hemoglobin** (g.100ml^{-1})	[11.5 - 17.5]	13.1±0.2	12.8±0.2
**Hematocrit** (%)	[34 - 53]	39±1	38±1
**MCV** - mean corpuscular volume (fL)	[76 - 96]	92±1	92±1
**MCH** - Mean corpuscular hemoglobin (pg)	[24.4 - 34]	30.7±0.2	30.9±0.4
**MCH content** (g.dL^{-1})	[31 - 36]	33.4±0.1	33.4±0.1
**WBC** - white blood cells (10^9.L^{-1})	[3.8 - 11]	5.84±0.28	6.53±0.25
**Neutrophils** (10^6.L^{-1})	[1400 - 7700]	2683±192	3528±202
**Eosinophils** (10^6.L^{-1})	[20 - 580]	213±31	168±29
**Basophils** (10^6.L^{-1})	[0 - 110]	37±4	38±4
**Lymphocytes** (10^6.L^{-1})	[1000 - 4800]	2369±114	2211±94
**Monocytes** (10^6.L^{-1})	[150 - 1000]	522±28	569±32
**Platelets** (10^9.L^{-1})	[150 - 445]	236±14	242±12
**Reticulocytes** (10^9.L^{-1})	[25 - 100]	52±5	43±5
**Prothrombin** (%)	[70 - 150]	106±2	95±2
**INR** – International Normalized Ratio	[0.85 - 1.35]	0.97±0.01	1.04±0.01
**aPTT** – activated partial thromboplastin time (sec.)	[21 - 35]	29.6±0.6	28±0.5
**PT ratio** - prothrombin time ratio (%)	[0 - 1.2]	0.98±0.02	0.93±0.02
**Fibrinogen** (g.L^{-1})	[2.38 - 4.98]	2.56±0.08	2.59±0.06
**Sodium** (mEq.L^{-1})	[136 - 145]	140±0	140±0
**Potassium** (mEq.L^{-1})	[3.5 - 5.1]	4.09±0.07	4.04±0.04
**Chlorine** (mEq.L^{-1})	[98 - 107]	108±0	107±0
**Alkaline reserve** (mEq.L^{-1})	[20 - 31]	27±0	27±0
**Proteins** (g.L^{-1})	[57 - 82]	66±1	66±1
**Albumin** (g.L^{-1})	[28 - 48]	42±0	42±0
**Urea** (g.L^{-1})	[0.19 - 0.49]	0.27±0.01	0.27±0.01
**Creatinine** (mg.L^{-1})	[5 - 11]	7.23±0.23	6.97±0.24

**Glucose** (g.L^{-1})	[0.74 - 1.06]	0.87±0.01	0.84±0.02
**Total bilirubin** (mg.L^{-1})	[3 - 12]	5.83±0.5	6.56±0.38
**Calcium** (mg.L^{-1})	[83 - 106]	90±1	89±0
**Phosphorus** (mg.L^{-1})	[24 - 51]	43±1	39±1
**AST** - aspartate transaminase (IU.L^{-1})	[13 - 40]	16±1	21±3
**ALT** - alanine transaminase (IU.L^{-1})	[7 - 40]	17±1	21±2
**CPK** - creatine phosphokinase (IU.L^{-1})	[34 - 145]	76±7	145±81
**ALP** - alkaline phosphatase (IU.L^{-1})	[46 - 116]	58±4	57±4
**GGT** - gamma glutamyl transferase (IU.L^{-1})	[0 - 38]	14±1	14±1

Supplementary Table.3 | Water balance and Urinary chemistry

Data are mean ± s.e.m. (n=18). One-way RM ANOVA with post-hoc Bonferroni multiple-comparison. *P<0.05 compared with B1. Shading: DI period.

	B2	B1	D1	D2	D3	D4	D5	R0	R1	ANOVA P value
**Water intake** (g)	3340±140	3600±180	**2750±120***	**2850±130***	**2740±120***	**2960±120***	**2870±70***	3740±170	3680±160	<0.0001
**Diuresis** (g)	2420±140	2750±170	2930±150	2710±140	**2290±100***	2530±120	2430±100	**2200±140***	2600±160	0.0001
**Partial water balance** (g)	920±50	860±50	**-180±60***	**140±100***	**460±50***	**430±50***	**440±40***	**1540±40***	1080±80	<0.0001
**Sodium** (mmol.24h^{-1})	**100±6***	151±8	**216±12***	143±7	**96±3***	**98±4***	**97±5***	**70±5***	**87±6***	0.007
**Potassium** (mmol.24h^{-1})	**86±7***	127±12	**182±23***	**112±11***	**76±4***	**85±4***	**82±6***	**60±5***	**74±7***	<0.0001
**Urinary Na^+/K^+ ratio**	1.29±0.13	1.4±0.18	2.04±0.44	1.51±0.18	1.33±0.09	1.2±0.09	1.22±0.06	1.27±0.09	1.23±0.05	0.09
**Chlorine** (mmol.24h^{-1})	**59±7***	82±9	92±13	80±11	71±6	69±7	67±4	63±3	64±6	0.007
**Osmolality** (mOsm.Kg^{-1})	252±13	274±13	287±11	283±12	281±13	253±11	270±13	301±14	270±20	0.122
**Urea** (mmol.24h^{-1})	255±15	282±13	240±21	313±12	290±12	290±10	307±16	**329±17***	355±31	0.004
**Creatinine** (mmol.24h^{-1})	9.2±0.4	10±0.3	9.6±0.5	10.1±0.3	10±0.2	9.4±0.3	9.1±0.4	9.8±0.3	11±0.8	0.15
**Urinary free cortisol** (µg.24h^{-1})	27±3	34±4	38±5	38±4	37±4	30±3	32±5	34±4	33±6	0.24
**Cortisol to creatinine ratio** (µg.g^{-1})	26±3	29±3	36±4	34±4	33±3	28±3	31±4	31±4	28±5	0.25
**Antidiuretic hormone** (pmol.24h^{-1})		23±3	23±2		28±4		19±1	24±2	22±3	0.12
**Total urinary nitrogen** (g.24h^{-1})		9.1±0.3					**10.8±0.5***			<0.0001 (paired T-test)

Supplementary Table.4 | Blood assessment
Data are mean ± s.e.m. (n=18) One-way RM ANOVA with post-hoc Bonferroni multiple-comparison. *P<0.05 compared with B1. Shading: DI period.

	**B1**	**D1evening**	**D3**	**D5**	**ANOVA P value**
**Complete blood count**					
**RBC** - red blood cells (10^{12}.L^{-1})	4.30±0.05	**4.51±0.06***	**4.91±0.06***	**4.82±0.07***	<0.0001
**Hemoglobin** (g.100ml^{-1})	12.8±0.2	**13.8±0.2***	**15.2±0.2***	**14.9±0.2***	<0.0001
**Hematocrit** (%)	39±1	**42±1***	**45±1***	**44±1***	<0.0001
**MCV** - mean corpuscular volume (fL)	92±1	92±1	92±1	92±1	0.38
**MCH** - Mean corpuscular hemoglobin (pg)	30.8±0.3	30.8±0.2	30.9±0.2	30.9±0.2	0.55
**MCH content** (g.dL^{-1})	33.6±0.1	33.5±0.1	33.5±0.1	33.6±0.1	0.82
**WBC** - white blood cells (10^{9}.L^{-1})	5.90±0.26	**7.59±0.4***	**6.81±0.3***	**7.09±0.39***	<0.0001
**Neutrophils** (10^{6}.L^{-1})	2975±194	**4361±337***	**4022±249***	**4190±358***	<0.0001
**Eosinophils** (10^{6}.L^{-1})	182±29	166±25	177±26	196±37	0.48
**Basophils** (10^{6}.L^{-1})	43±5	42±5	41±5	43±5	0.76
**Lymphocytes** (10^{6}.L^{-1})	2215±112	2337±90	**2053±102***	2123±124	0.03
**Monocytes** (10^{6}.L^{-1})	477±29	**598±42***	499±32	518±36	0.0006
**Platelets** (10^{9}.L^{-1})	243±15	257±16	**272±16***	**265±16***	0.0008
**Reticulocytes** (10^{9}.L^{-1})	50±5	55±5	60±6	60±5	0.13
**Hemostasis**					
**PT ratio** - prothrombin time ratio (%)	0.98±0.02	0.99±0.03	1.02±0.02	1.00±0.02	0.12
**aPTT** – activated partial thromboplastin time (sec.)	29.6±0.5	29.7±0.8	30.6±0.5	30.1±0.5	0.22
**Fibrinogen** (g.L^{-1})	2.53±0.08	2.51±0.07	**2.93±0.07***	**2.96±0.08***	<0.0001
**Chemistry panel & Inflammatory state**					
**Sodium** (mmol.L^{-1})	140.9±0.4	140.1±0.2	**138.8±0.2***	**138.9±0.3***	0.0001
**Potassium** (mmol.L^{-1})	3.94±0.04	3.88±0.07	**4.16±0.04***	4.02±0.04	0.003
**Chlorine** (mmol.L^{-1})	105.9±0.5	105±0.41	**103.72±0.29***	**103.39±0.34***	<0.0001
**Osmolality** (mOsmol.kg^{-1})	287±2	290±2	286±1	285±1	0.1
**Proteins** (g.L^{-1})	69±1	**72±1***	**76±1***	**74±1***	<0.0001
**Albumin** (g.L^{-1})	44±1	47±1	**48±1***	46±1	0.009
**Urea** (mmol.L^{-1})	3.3±0.1	3.2±0.1	3.4±0.2	3.4±0.2	0.32
**Creatinine** (µmol.L)	60±2	**56±2***	58±2	60±2	0.001
**AST** (IU.L^{-1})	17±1	**19±1***	19±1	**20±1***	0.014
**ALT** (IU.L^{-1})	11±1	11±1	11±1	13±2	0.23
**hs-CRP** (mg.L^{-1})	0.5±0.1	0.5±0.1	0.5±0.1	0.6±0.1	0.71
**Metabolic markers**					
**Triglycerides** (mmol.L^{-1})	0.7±0.04		**0.94±0.07***	**0.96±0.05***	<0.0001
**Total cholesterol** (mmol.L^{-1})	4.26±0.16		**4.88±0.15***	**4.67±0.15***	<0.0001
**HDL cholesterol** (mmol.L^{-1})	1.59±0.08		**1.68±0.08***	1.56±0.08	0.0006
**LDL cholesterol** (mmol.L^{-1})	2.4±0.1		**2.8±0.1***	**2.7±0.1***	<0.0001
**AIP**	-0.36±0.04		**-0.26±0.04***	**-0.21±0.03***	<0.0001
**Fasting glucose** (mmol.L^{-1})	4.67±0.06		**4.89±0.07***	4.59±0.05	0.0002
**Fasting insulin** (µIU.L^{-1})	7.5±0.6		**10.1±0.8***	8.4±0.7	<0.0001
**HOMA-IR**	1.56±0.13		**2.20±0.19***	1.72±0.14	0.0001
**Adiponectin** (µg.mL^{-1})	9.9±1		9.9±1	**8.3±0.8***	<0.0001
**NEFA** (µmol.L^{-1})	252±28			**334±31***	0.007 (paired T-test)
**Hormones**					
**Renin** (ng.L^{-1})	6.6±0.9	**2.9±0.7***	**12.7±1.4***	**20.6±2.6***	<0.0001
**Aldosterone** (ng.L^{-1})	108±15	63±8	219±34	278±38	<0.0001

**BNP** (ng.L^{-1})	14.1±1.9	**27.1±3.8***	**8.5±1.8***	**5.4±1.3***	<0.0001
**Testosterone** (nmol.L^{-1})	0.88±0.07		**1.07±0.10***	1.02±0.08	0.025
**Free T4** (pmol.L^{-1})	12.8±0.3		**15.2±0.4***	**15.1±0.4***	<0.0001
**TSH** (mU.L^{-1})	1.4±0.1		**1.9±0.2***	**1.9±0.2***	<0.0001
**Soluble markers of endothelial state**					
**VEGF** (pg.mL^{-1})	153±26		**177±136***	185±166	0.04
**VEGFR-1** (pg.mL^{-1})	295±11		**388±57***	**338±66***	<0.0001
**E-Selectin** (ng.mL^{-1})	28±2		**31±10***	**30±11***	0.0002
**Globulins**					
**Alpha1 globulins** (%)	3.63±0.11		**3.58±0.1**	3.54±0.1	0.3
**Alpha1 globulins** (g.L^{-1})	2.39±0.09		**2.65±0.08***	2.51±0.08	0.0004
**Alpha2 globulins** (%)	8.47±0.19		**9.04±0.18***	**8.88±0.15***	<0.0001
**Alpha2 globulins** (g.L^{-1})	5.61±0.16		**6.67±0.14***	**6.27±0.15***	<0.0001
**Beta1 globulins** (%)	6.27±0.14		**6.18±0.14***	**6.16±0.15***	0.006
**Beta1 globulins** (g.L^{-1})	4.15±0.13		**4.56±0.13***	**4.35±0.15***	<0.0001
**Beta2 globulins** (%)	4.79±0.25		**4.98±0.26***	4.94±0.25	0.01
**Beta2 globulins** (g.L^{-1})	3.18±0.19		**3.69±0.2***	**3.57±0.23***	0.0004
**Gamma globulins** (%)	16±0.5		**16.3±0.5***	16.1±0.5	<0.0001
**Gamma globulins** (g.L^{-1})	10.6±0.4		**12.1±0.4***	**11.4±0.4***	<0.0001

Supplementary Table.5 | Serum markers for bone turnover and bone metabolism regulation
Data are mean ± s.e.m. (n=18) One-way RM ANOVA with post-hoc Bonferroni multiple-comparison. *P<0.05 compared with B mean. Shading: DI period.

	**B2**	**B1**	**B** mean	**D2**	**D3**	**R0**	**R2**	**ANOVA P value**
	**Resorption activity**							
**CTx** (pmol.L^{-1})	5070±600	4996±574	5033±584	4721±467	5211±528	5596±504	4908±468	0.0005
	**Formation activity**							
**P1NP** (µg.L^{-1})	81±8	83±9	82±9	**72±8***	78±8	**66±7***	**68±7***	<0.0001
**Intact OC** (ng.mL^{-1})	25.2±2.2	23.9±2.1	24.5±2.1	**22.7±2.1***	**23.2±2***	**21.9±1.6***	**20.7±1.6***	0.0004
**Gla-OC** (ng.mL^{-1})	10.8±0.8	10.1±0.8	10.5±0.8	**9.4±0.7***	**9.7±0.7***	**8.9±0.6***	**8.7±0.6***	<0.0001
**Glu-OC** (ng.mL^{-1})	9.9±1.3	9.8±1.2	9.9±1.2	9.6±1.1	10.6±1.2	9.8±1	**7.7±0.9***	<0.0001
**Phosphocalcic metabolism and its regulation**								
**Calcium** (mg.L^{-1})	94±1	95±1	94±1	**97±1***	**99±1***	**97±1***	**93±1***	<0.0001
**Phosphorus** (mg.L^{-1})	43.7±0.9	41.2±0.9	42.5±0.8	41.6±0.8	44.1±1	**40.2±0.6***	**39.1±0.6***	<0.0001
**25 OH-D** (ng.mL^{-1})	26.3±1.2	26.4±1.3	26.3±1.3	26±1.3	26.6±1.4	26.3±1.4	25.3±1.4	0.009
**PTH** (ng.L^{-1})	28±1	28±1	28±1	26±1	**25±1***	**23±2***	24±2	0.003

Supplementary Table.6 | Caloric and dietary intake

Meals of each experiment day were identical for all participants (n=18), and dietary intake was individually tailored (standardized to body weight in energy and nutrients) and controlled during the study. Data are mean ± s.e.m. Shading indicates DI period.

	B4	B3	B2	B1	D1	D2	D3	D4	D5	R0	R1
**Energy** (Kcal)	2109 ± 43	2116 ± 39	2122 ± 40	2123 ± 40	1729 ± 32	1728 ± 32	1695 ± 39	1728 ± 32	1728 ± 32	2116 ± 40	2090 ± 54
**CarboHydrate** (g)	255.7 ± 6.1	255.1 ± 5.3	264.5 ± 4.4	265.2 ± 5.5	193.5 ± 4.6	206.3 ± 4.9	191.4 ± 6.7	202.0 ± 4.4	202.6 ± 4.6	261.9 ± 5.6	248.8 ± 6.9
**Protein** (g)	67.4 ± 1.6	68.5 ± 1.3	66.1 ± 1.6	66.4 ± 1.3	67.5 ± 1.3	63.7 ± 1.1	63.6 ± 1.4	62.6 ± 1.1	61.6 ± 1.2	68.0 ± 1.4	69.4 ± 2.2
**Fat** (g)	81.7 ± 1.8	85.8 ± 1.7	81.1 ± 1.9	81.1 ± 1.6	69.7 ± 1.4	65.5 ± 1.2	65.1 ± 1.6	69.2 ± 1.4	67.4 ± 1.4	81.1 ± 1.7	84.2 ± 2.3
**SFA** - saturated fatty acid (g)	29.3 ± 0.7	23.7 ± 0.5	20.0 ± 0.5	28.6 ± 0.5	19.1 ± 0.4	21.5 ± 0.4	22.8 ± 0.6	19.9 ± 0.4	18.4 ± 0.5	29.1 ± 0.5	21.4 ± 0.6
**MFA** - monounsaturated fatty acid (g)	27.9 ± 0.7	26.8 ± 0.6	27.1 ± 1.0	26.7 ± 0.9	25.4 ± 0.6	21.8 ± 0.4	23.2 ± 0.7	21.3 ± 0.4	22.0 ± 0.5	26.7 ± 0.7	32.0 ± 1.2
**PUFA** - polyunsaturated fatty acid (g)	18.3 ± 0.4	27.4 ± 0.7	21.7 ± 0.6	15.9 ± 0.4	14.1 ± 0.5	16.7 ± 0.4	14.4 ± 0.6	21.1 ± 0.7	16.5 ± 0.4	15.0 ± 0.6	18.9 ± 0.6
**Total Water** (g)	3228 ± 135	3354 ± 144	3339 ± 142	3601 ± 176	4303 ± 259	4404 ± 271	2741 ± 125	2960 ± 123	2870 ± 75	3735 ± 169	5234 ±235
**Na** - sodium (mg)	3395 ± 81	3034 ± 52	3639 ± 68	4885 ± 74	3045 ± 54	3262 ± 78	2706 ± 68	2777 ± 64	3089 ± 53	4895 ± 99	3398 ± 97
**Cl** - chloride (mg)	5590 ± 123	4819 ± 68	5789 ± 106	7638 ± 103	4778 ± 74	3958 ± 84	4257 ± 108	4347 ± 95	5021 ± 93	7601 ± 150	5122 ± 149
**K** - potassium (mg)	3713 ± 62	4101 ± 70	2953 ± 56	4347 ± 103	2790 ± 46	2823 ± 62	2795 ± 60	3634 ± 87	2947 ± 53	4286 ± 94	3047 ± 58
**Ca** - calcium (mg)	2084 ± 64	2177 ± 61	2210 ± 65	2288 ± 83	1888 ± 62	2011 ± 68	1798 ± 68	1943 ± 64	1878 ± 42	2383 ± 104	2253 ± 85
**Fibers** (g)	34.9 ± 0.7	32.0 ± 0.6	21.7 ± 0.6	34.4 ± 0.8	26.3 ± 0.5	25.3 ± 0.6	19.3 ± 0.5	26.9 ± 0.7	24.2 ± 0.6	34.1 ± 0.7	32.4 ± 0.7
**Vitamine A** (µg)	841 ± 19	1911 ± 55	808 ± 13	3026 ± 45	886 ± 26	2366 ± 116	726 ± 20	1716 ± 89	686 ± 17	2839 ± 86	893 ± 27
**Vitamine K** (µg)	215 ± 6	329 ± 8	338 ± 5	358 ± 7	416 ± 12	368 ± 10	197 ± 6	273 ± 7	306 ± 5	355 ± 9	394 ± 12
**Vitamine C** (mg)	403 ± 8	159 ± 4	103 ± 2	306 ± 11	203 ± 4	85 ± 2	233 ± 10	140 ± 4	106 ± 2	304 ± 7	221 ± 5
**Vitamine B1** (mg)	2.24 ± 0.04	1.99 ± 0.03	1.04 ± 0.03	1.50 ± 0.00	1.24 ± 0.02	1.07 ± 0.03	1.7 ± 0.04	1.7 ± 0.03	1.02 ± 0.02	1.5 ± 0.03	1.41 ± 0.03
**Vitamine B2** (mg)	1.77 ± 0.03	1.58 ± 0.02	1.31 ± 0.03	1.60 ± 0.00	1.51 ± 0.03	1.52 ± 0.03	1.42 ± 0.03	1.42 ± 0.03	1.24 ± 0.02	1.65 ± 0.03	1.59 ± 0.05
**Vitamine B3** (mg)	24.3 ± 0.6	30.3 ± 0.8	25.5 ± 0.7	28.5 ± 0.5	28.6 ± 0.5	25.6 ± 0.5	22.5 ± 0.6	28.3 ± 0.8	25.3 ± 0.5	28.9 ± 0.7	29.2 ± 0.8
**Vitamine B5** (mg)	6.03 ± 0.10	6.26 ± 0.10	4.25 ± 0.09	5.10 ± 0.10	4.52 ± 0.07	4.96 ± 0.07	5.00 ± 0.10	5.70 ± 0.13	3.90 ± 0.06	5.17 ± 0.09	4.71 ± 0.12
**Vitamine B6** (mg)	1.98 ± 0.04	2.41 ± 0.04	1.76 ± 0.05	2.30 ± 0.00	1.53 ± 0.02	2.04 ± 0.04	1.47 ± 0.03	2.14 ± 0.04	1.71 ± 0.03	2.26 ± 0.04	1.62 ± 0.04
**Vitamine B8** (µg)	32.6 ± 1.0	53.6 ± 0.8	36.0 ± 0.8	38.2 ± 0.9	31.8 ± 0.7	42.0 ± 0.8	23.5 ± 0.6	46.1 ± 0.9	30.8 ± 0.8	38.0 ± 0.8	38.4 ± 1.2
**Vitamine B9** (µg)	414 ± 8	486 ± 9	326 ± 7	345 ± 10	373 ± 7	326 ± 8	295 ± 9	440 ± 15	292 ± 5	339 ± 8	409 ± 12
**Vitamine B12** (µg)	2.42 ± 0.06	3.30 ± 0.11	2.05 ± 0.06	3.10 ± 0.10	2.99 ± 0.08	2.54 ± 0.06	2.69 ± 0.05	3.39 ± 0.11	1.73 ± 0.04	3.23 ± 0.09	2.82 ± 0.13
**Vitamine D** (µg)	1.17 ± 0.03	2.50 ± 0.07	1.39 ± 0.04	2.20 ± 0.0	1.46 ± 0.03	1.86 ± 0.04	0.79 ± 0.02	2.46 ± 0.07	1.22 ± 0.03	2.17 ± 0.05	1.39 ± 0.05
**Vitamine E** (mg)	19.5 ± 0.5	24.1 ± 0.5	19.1 ± 0.5	18.6 ± 0.6	16.6 ±0.5	13.0 ± 0.3	15.6 ± 0.3	19.0 ± 0.5	17.2 ± 0.3	18.1 ± 0.5	22.5 ± 0.7
**Mg** - magnesium (mg)	601 ± 32	611 ± 35	630 ± 36	621 ± 40	451 ± 26	518 ± 30	529 ± 27	553 ± 31	510 ± 26	688 ± 37	700 ± 46
**P** - phosphorus (mg)	973 ± 18	1367 ± 21	1068 ± 24	1194 ± 26	1081 ± 20	1144 ± 19	850 ± 17	1261 ± 22	967 ± 19	1225 ± 25	1118 ± 36
**Fe** - iron (mg)	10.3 ± 0.2	15.5 ± 0.3	9.4 ± 0.2	12.5 ± 0.3	9.3 ± 0.2	8.8 ± 0.2	7.8 ± 0.1	14.0 ± 0.3	8.7 ± 0.2	12.3 ± 0.3	10.6 ± 0.2
**Cu** - copper (mg)	1.69 ± 0.03	1.92 ± 0.03	1.52 ± 0.03	2.40 ± 0.10	1.31 ± 0.02	1.54 ± 0.03	1.24 ± 0.02	1.62 ± 0.03	1.40 ± 0.03	2.34 ± 0.06	1.53 ± 0.04
**Zn** - zinc (mg)	13.1 ± 0.3	11.7 ± 0.2	9.6 ± 0.2	11.7 ± 0.3	10.3 ± 0.2	9.1 ± 0.2	11.3 ± 0.2	10.0 ± 0.2	8.7 ± 0.2	11.9 ± 0.2	11.6 ± 0.3
**Id** - iodine (µg)	121 ± 3	279 ± 8	110 ± 3	241 ± 6	104 ± 2	102 ± 2	71 ± 2	271 ± 10	105 ± 3	235 ± 7	120 ± 3

Supplementary Table.7 | Complete list of assays and methods for blood and urine assessment

Assay	Method - kit - analyzer
**BLOOD**	
**Hemoglobin**	HemoCue Hb-201®
**Hematocrit**	Microcentrifugation
**Complete blood count & Hemostasis**	Automated Hematology & Coagulation analyzers
**Electrolytes (Na+, K+, Cl−)**	Indirect potentiometry - Advia chemistry XPT (Siemens)
**Osmolality**	Freezing-point depression in comparison to pure water
**Total protein**	Biuret - Advia chemistry XPT (Siemens)
**Albumin**	Immuno-turbidimetry (Diagam) sur Advia chemistry XPT (Siemens)
**Urea**	Urease technique - Advia chemistry XPT (Siemens)
**Creatinine**	Enzymatic method - Advia chemistry XPT (Siemens)
**Glucose**	Hexokinase - Advia chemistry XPT (Siemens)
**AST; ALT**	IFCC method - Advia chemistry XPT (Siemens)
**hs-CRP**	Immuno-turbidimetry - Advia chemistry XPT (Siemens)
**Triglycerides**	Enzymatic technique - Advia chemistry XPT (Siemens)
**Total cholesterol**	Cholesterol oxydase - Advia chemistry XPT (Siemens)
**HDL cholesterol**	Direct technique/catalase-elimination - Advia chemistry XPT (Siemens)
**LDL cholesterol**	Friedewald formula
**Serum protein electrophoresis**	Capillaris (Sebia)
**Insulin**	Chemiluminescence - Advia Centaur XPT (Siemens)
**Adiponectin**	ELISA BioVendor
**NEFA** (non-esterified fatty acids)	Enzymatic colorimetric assay (Pentra Horriba)
**Renin**	Chemiluminescence immunoassay, Liaison Analyzer (DiaSorin)
**Aldosterone**	Competition radioimmunoassay, RIA kit (Immunotech, Beckman Coulter)
**BNP**	Chemiluminescence immunoassay, Architect i2000 Analyzer (Abbott)
**Estradiol**	Competitive radioimmunoassay, RIA kit (Immunotech, Beckman Coulter)
**Progesterone; Testosterone; Free T4; TSH**	Chemiluminescence - Advia Centaur XPT (Siemens)
**VEGF**	Human VEGF Quantikine ELISA kit
**VEGFR-1**	Human VEGFR1/Flt-1 Quantikine ELISA kit
**E-selectin**	Human sE-Selectin/CD62E Quantikine ELISA kit
**CTx; P1NP; Intact and N-mid osteocalcin fragment (OC)**	Chemiluminescence immunoassay (IDS-iSYS automated analyzer, Boldon, UK)
**Glu-OC; Gla-OC**	Enzyme-immunoassay (EIA) kits (Takara Bio, Inc., Otsu, Japan)
**Serum intact PTH; calcium; phosphorus**	Electrochemiluminescence immunoassay (Cobas®8000 modular analyzer, Roche Diagnostics Ltd., Rotkreuz, Switzerland)
**25 OH-D**	Chemiluminescence immunoassay (Architect i2000SR chemiflex analyzer, Abbott Park, Illinois, USA)
**URINE**	
**Electrolytes (Na^+, K^+, Cl^-)**	Indirect potentiometry - Advia chemistry XPT (Siemens)
**Osmolality**	Freezing-point depression in comparison to pure water
**Urea; Creatinine**	Advia chemistry XPT (Siemens)
**Total urinary nitrogen**	Automated Dumas technique system
**Urinary free cortisol**	Liquid chromatography-tandem mass spectrometry, Applied Biosystems/MDS Sciex Api 3000 triple quad mass spectrometer
**Antidiuretic hormone (ADH)**	Radioimmunoassay (Diasource)

Supplementary figures

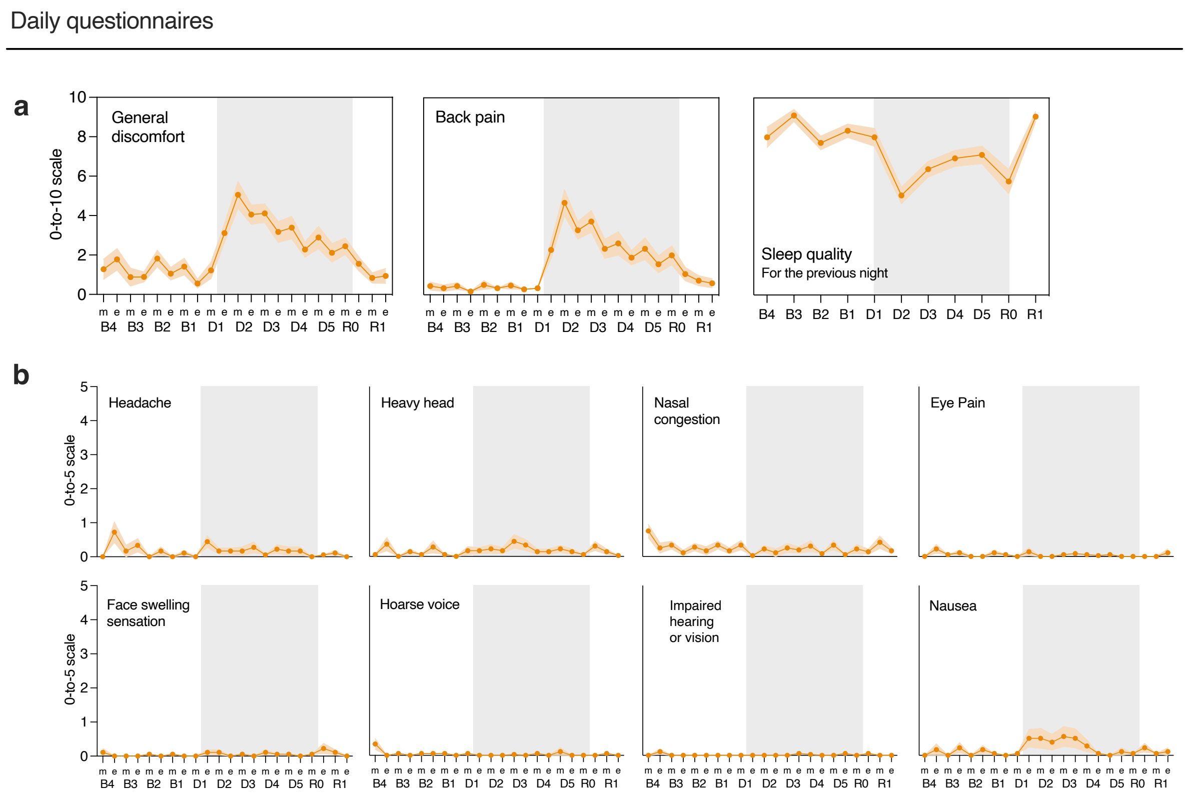

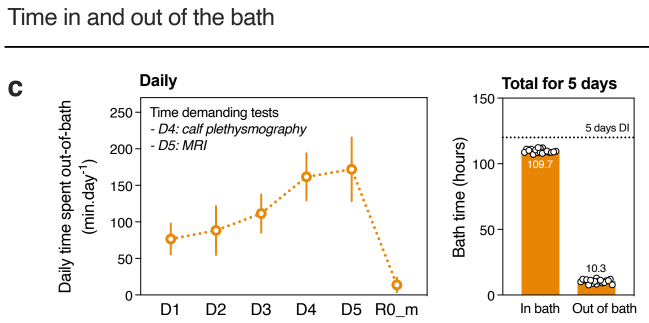

d

Urination option	In-bath time (h)
Purewick™ only (n=3)	110.7±1.9
Purewick™ and urinary basin (n=3)	110.7±1.8
Urinary basin only (n=12)	109.2±1.2

Supplementary Fig.1 | Tolerance of DI and out-of-bath time
a, General discomfort and Back pain (morning (m) and evening (e) assessment), and Quality of night sleeping (morning assessment) using 0-to-10 visual analog scale (n=18). **b**, Complaints related to fluid shift: headache, heavy head, nasal congestion, eye pain, face swelling sensation, hoarse voice, impaired hearing or vision, and nausea; morning (m) and evening (e) assessment using 0-to-5 score (n=18). **c**, Out-of-bath time for each day of DI, and Total time spent in- and out-of-bath during 5 days (120h) of DI (n=18). **d**, Urination option and related time spent in bath. The Purewick™ system, primarily designed for women suffering from urinary incontinence, aspirates urine from the soft flexible wick (external catheter) to a sealed collector. **a**, **b**, **c**, **d**, Data are mean ± s.e.m. **d**, Data are mean ± SD. Shading indicates DI period.

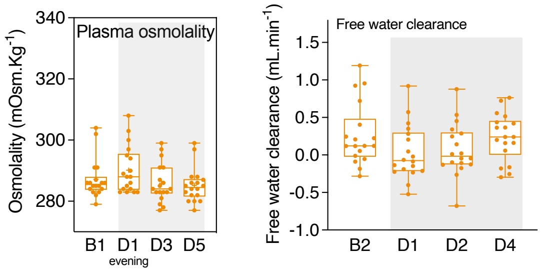

Supplementary Fig.2 | Plasma osmolality and free water clearance.
Box plots indicate minimum, 25th percentile, median, 75th percentile and maximum (n=18). One-way RM ANOVA compared with baseline. Global ANOVA results: Plasma osmolality P=0.093, free water clearance P=0.176. Shading indicates DI period.

Hormones and menstrual cycle

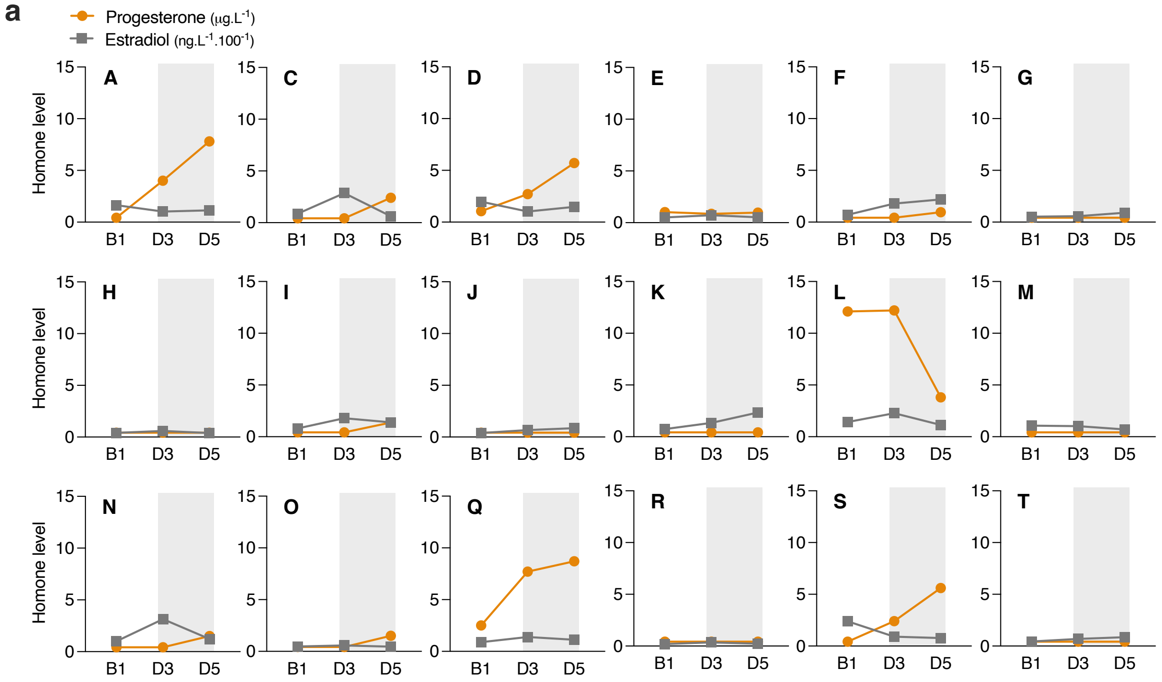

b

Ovulation ▼

	1	2	3	4	5	6	7	8	9	10	11	12	13	14	15	16	17	18	19	20	21	22	23	24	25	26	27	28
A										B3	B2	B1	D1	D2	D3	D4	D5	R0	R1	R2								
C								B3	B2	B1	D1	D2	D3	D4	D5	R0	R1	R2										
D													B3	B2	B1	D1	D2	D3	D4	D5	R0	R1	R2					
E			B3	B2	B1	D1	D2	D3	D4	D5	R0	R1	R2															
F						B3	B2	B1	D1	D2	D3	D4	D5	R0	R1	R2												
G				B3	B2	B1	D1	D2	D3	D4	D5	R0	R1	R2														
H		B3	B2	B1	D1	D2	D3	D4	D5	R0	R1	R2																
I							B3	B2	B1	D1	D2	D3	D4	D5	R0	R1	R2											
J					B3	B2	B1	D1	D2	D3	D4	D5	R0	R1	R2													
K					B3	B2	B1	D1	D2	D3	D4	D5	R0	R1	R2													
L																B3	B2	B1	D1	D2	D3	D4	D5	R0	R1	R2		
M						B3	B2	B1	D1	D2	D3	D4	D5	R0	R1	R2												
N							B3	B2	B1	D1	D2	D3	D4	D5	R0	R1	R2											
O							B3	B2	B1	D1	D2	D3	D4	D5	R0	R1	R2											
Q																B3	B2	B1	D1	D2	D3	D4	D5	R0	R1	R2		
R		B3	B2	B1	D1	D2	D3	D4	D5	R0	R1	R2																
S										B3	B2	B1	D1	D2	D3	D4	D5	R0	R1	R2								
T			B3	B2	B1	D1	D2	D3	D4	D5	R0	R1	R2															

Supplementary Fig.3 | Hormones level and individual menstrual cycle phase during protocol (as estimated from blood progesterone & estradiol level)
a, Individual level of progesterone and estradiol from morning blood sampling. **b**, Table of menstrual cycle phase estimated from the hormones level. Shading indicates DI period.
