# Supplementary material for: Comprehensive assessment of physiological responses in women during the ESA dry immersion VIVALDI microgravity simulation
Source: Nat Commun. 2023 Oct 9;14:6311. doi: 10.1038/s41467-023-41990-4 (PMC10562467; doi:10.1038/s41467-023-41990-4)
Supplement: Supplementary file 3 — Reporting Summary [file 41467_2023_41990_MOESM3_ESM.pdf]

nature portfolio

Corresponding author(s): Adrien Robin

Last updated by author(s): Sep 5, 2023

Reporting Summary

Nature Portfolio wishes to improve the reproducibility of the work that we publish. This form provides structure for consistency and transparency in reporting. For further information on Nature Portfolio policies, see our Editorial Policies and the Editorial Policy Checklist.

Statistics

For all statistical analyses, confirm that the following items are present in the figure legend, table legend, main text, or Methods section.

n/a	Confirmed	
☐	☒	The exact sample size (*n*) for each experimental group/condition, given as a discrete number and unit of measurement
☒	☐	A statement on whether measurements were taken from distinct samples or whether the same sample was measured repeatedly
☐	☒	The statistical test(s) used AND whether they are one- or two-sided<br>*Only common tests should be described solely by name; describe more complex techniques in the Methods section.*
☒	☐	A description of all covariates tested
☐	☒	A description of any assumptions or corrections, such as tests of normality and adjustment for multiple comparisons
☐	☒	A full description of the statistical parameters including central tendency (e.g. means) or other basic estimates (e.g. regression coefficient) AND variation (e.g. standard deviation) or associated estimates of uncertainty (e.g. confidence intervals)
☐	☒	For null hypothesis testing, the test statistic (e.g. *F*, *t*, *r*) with confidence intervals, effect sizes, degrees of freedom and *P* value noted<br>*Give P values as exact values whenever suitable.*
☒	☐	For Bayesian analysis, information on the choice of priors and Markov chain Monte Carlo settings
☒	☐	For hierarchical and complex designs, identification of the appropriate level for tests and full reporting of outcomes
☐	☒	Estimates of effect sizes (e.g. Cohen's *d*, Pearson's *r*), indicating how they were calculated

Our web collection on statistics for biologists contains articles on many of the points above.

Software and code

Policy information about availability of computer code

Data collection	Body composition (lean and fat mass, and bone mineral density) was performed by a QDR 4500W scanner using the version software 11.2 (Hologic, Nassy, France). Maximal oxygen uptake data during the aerobic capacity test were collected using LABManager 5.3.0.4 (Cardinal Health, Germany). The optic nerve sheath diameter was determind by ultrasonography using Ondina software v1.1.2 (Sonoscanner, Paris, France). Postural balance data during the postorography test were collected using Leonardo Mechanography v4.2 software (Novotec Medical, Switzerland). Maximal voluntary muscle contraction data were collected using Human Kinetics 1.7.5 software. The calf volume during the venous compliance test and hemodynamics parameters during the orthostatic tolerance test were collected through a Biopac device with AcqKnowledge software version 5.0 (BIOPAC systems, CA, USA). During the indirect calorimetry, oxygen consumption and carbon dioxyde excretion were collected using OMNIA 2.0 software (Cosmed, Italy). Physical activity data with accelerometers were collected using ActiLife5 software (Actigraph, FL, USA). Core body temperature data were collected using e-Performance manager 1.3.2 software (BodyCap, France). Continuous heart rate and blood pressure data were collected using Domino light 1.4 software (SOMNOmedics, Germany). Nailfold capillary density data were collected using DinoCapture 2.0 (Dino-lite Medical, France).
Data analysis	Our statistical analyses were conducted using GraphPad Prism 9.4.0

For manuscripts utilizing custom algorithms or software that are central to the research but not yet described in published literature, software must be made available to editors and reviewers. We strongly encourage code deposition in a community repository (e.g. GitHub). See the Nature Portfolio guidelines for submitting code & software for further information.

Data

Policy information about availability of data

All manuscripts must include a data availability statement. This statement should provide the following information, where applicable:

- Accession codes, unique identifiers, or web links for publicly available datasets
- A description of any restrictions on data availability
- For clinical datasets or third party data, please ensure that the statement adheres to our policy

DATA AVAILABILITY STATEMENT
The scientific community can request access to the raw data resulting from ESA's Dry Immersion studies for retrospective studies. ESA is committed to making this data available in order to promote scientific progress and knowledge sharing.
After data curation and handling, the data will be archived at the ESA HRE Data Archive, mandated by ESA’s Human and Robotic Exploration (HRE) Programs Directorate (link here: https://hreda.esac.esa.int/hreda/#/pages/home). Data will be made available by ESA, i.e., after a request has been made and dully justified. The latter is in line with ESA’s Personal Data Protection Framework rules (https://esamultimedia.esa.int/docs/LEX-L/ESA_Principles_of_PDP_Rules_of_Procedure_for_DPSA_and_Policy.pdf). The entire protocol, as submitted for regulatory approvals, will be available too on the ESA HRE Data Archive website.

Research involving human participants, their data, or biological material

Policy information about studies with human participants or human data. See also policy information about sex, gender (identity/presentation), and sexual orientation and race, ethnicity and racism.

Reporting on sex and gender	Data and analysis presented in our study refers to biological women, the gender was not discussed here. Information regarding disaggregated sex and gender data has not been collected.
Reporting on race, ethnicity, or other socially relevant groupings	NA
Population characteristics	Baseline general details of the population characteristics are included in main text, full caloric and dietary intake are included in Supplementary Table 6, full blood assessment at the begining of the study (performed by the clinic for medical safety) are included in Supplementary Table 1.
Recruitment	The call for candidates through advertisements via Internet and media started after regulatory approval. Selection consisted of preliminary screening, followed by medical examinations.<br>The inclusion criteria were: age 20 to 40 years old; body mass index (BMI) between 20 and 26 kg.m -2 ; no sedentary nor high-level athletes with VO2 max between 30 and 55 mL.kg -1 .min -1 ; height between 158 and 180 cm; no combined estroprogestative contraception (progestogen-only pills, intrauterine devices, implants or absence of contraception allowed); certified as healthy by a comprehensive clinical assessment including a detailed medical history and complete physical examination; covered by a Health insurance. The non-inclusion criteria were: any chronic disease; acute infection; cardiovascular, neurological (in particular vestibular disorders and orthostatic hypotension), ear-nose-throat, orthopedic or musculoskeletal disorders; tobacco, alcohol, or drug addiction; medications except for the accepted means of contraception.
Ethics oversight	The experimental protocol conformed to the standards set by the Declaration of Helsinki and was approved by the national Ethic Committee (CPP Ile de France II: July 5, 2021, n°ID RCB: 2021-A00705-36) and French Health Authorities (ANSM: May 31, 2021).

Note that full information on the approval of the study protocol must also be provided in the manuscript.

Field-specific reporting

Please select the one below that is the best fit for your research. If you are not sure, read the appropriate sections before making your selection.

☒ Life sciences ☐ Behavioural & social sciences ☐ Ecological, evolutionary & environmental sciences

For a reference copy of the document with all sections, see nature.com/documents/nr-reporting-summary-flat.pdf

Life sciences study design

All studies must disclose on these points even when the disclosure is negative.

Sample size	Power-based calculation of the number of subjects is not directly applicable for such explorative studies, so to determine sample size, we based on combined considerations of physiological assessments, taking into account already known effects of DI. Thus, based on effect sizes obtained with previous MEDES DI in men (Robin et al. 2020; De Abreu et al. 2017), minimal sample to find statistically significant difference between Pre- and Post-DI, when there is one (at power 80% and alpha level 0.05), could be estimated for example as n=5 for plasma volume evolution (effect size 2.01), n=11 for orthostatic tolerance (effect size 0.96), n=15 for glucose tolerance (effect size 0.8), n=19 for VO2max (effect size 0.69). Therefore a total of 20 subjects was deemed necessary for the study and approved by the national Ethic Committee.
Data exclusions	Optic Nerve Sheath Diameter was not clearly defined in one participant and was excluded. Calf plethysmography data were excluded for analysis for the first participant due to slight methodological changes (duration of venous occlusion steps) compared to the rest of the

participants. For orthostatic tolerance test, one participant presented acute bradychardia during the pre-DI test, and was not tested post-DI for safety reasons. Participants exclusions were specified in the manuscript.

Replication	No replication was performed
Randomization	All the participants were considered as a control group. No randomization was performed
Blinding	The investigators were not blinded, as only a control group was studied

Reporting for specific materials, systems and methods

We require information from authors about some types of materials, experimental systems and methods used in many studies. Here, indicate whether each material, system or method listed is relevant to your study. If you are not sure if a list item applies to your research, read the appropriate section before selecting a response.

Materials & experimental systems

n/a	Involved in the study
☒	☐ Antibodies
☒	☐ Eukaryotic cell lines
☒	☐ Palaeontology and archaeology
☒	☐ Animals and other organisms
☐	☒ Clinical data
☒	☐ Dual use research of concern
☒	☐ Plants

Methods

n/a	Involved in the study
☒	☐ ChIP-seq
☒	☐ Flow cytometry
☒	☐ MRI-based neuroimaging

Clinical data

Policy information about clinical studies
All manuscripts should comply with the ICMJEguidelines for publication of clinical research and a completedCONSORT checklist must be included with all submissions.

Clinical trial registration	ClinicalTrials.gov Identifier: NCT05043974.
Study protocol	Protocol availability: The entire protocol, as submitted for regulatory approvals, will be available on the ESA HRE Data Archive website.
Data collection	Data were collected during one monocentric campaign in the MEDES space clinic (Toulouse, France) from 20 Sept 2021 to 10 Dec 2021.

Outcomes

Outcome Measures :
1. Change in orthostatic tolerance [Time Frame: At baseline and after five days of dry immersion]
   - Orthostatic tolerance assessed during a Lower Body Negative Pressure test (LBNP test)
2. Change in peak aerobic power (VO2max test) [Time Frame: At baseline and after five days of dry immersion]
   - Exercise capacity assessed by graded cycling on sitting ergometer until exhaustion
3. Change in plasma volume [Time Frame: At baseline and during dry immersion]
   - Plasma volume (L) assessed by the Dill & Costill method
4. Change in fluid shift distribution towards the cephalic region [Time Frame: At baseline, the first day to quantify the short term effect and the fifth day of dry-immersion to quantify the long term effect of fluid shift]
   - The hormones involved in fluid distribution assessed in blood and urine samples
5. Change in vascular endothelium integrity [Time Frame: At baseline and during the five days of the dry-immersion period]
   - Vascular endothelium integrity assessed by blood parameters of vascular and endothelial integrity.
6. Change in circadian rhythms of blood pressure [Time Frame: At baseline and during the five days of the dry-immersion period]
   - Continuous 24-h recording of blood pressure performed by SOMNOtouch™ NIBP system designed for ambulatory continuous measurements
7. Change in lower limb veins functions [Time Frame: At baseline, after four days of dry-immersion and after one day of recovery]
   - Venous compliance of lower limbs assessed by plethysmography.
8. Change in body fluid compartments by bioelectrical impedance analysis [Time Frame: Baseline and during five days of dry-immersion]
   - Extracellular, intracellular and total body water estimated by bioimpedance
9. Change in muscle strength [Time Frame: At baseline and after five days of dry-immersion]
   - Muscle strength assessed from single leg isometric maximal voluntary contraction on the knee extensors & flexors, the plantarflexors and dorsiflexors. The Isometric Torque will be measured in Nm. The peak of the three maximal attempts recorded for strength measures.
10. Change in muscle volume at calf level [Time Frame: At baseline and after five days of dry-immersion]
   - Muscle dehydration, eventual atrophy and fatty degeneration measured by quantitative Dixon MRI sequences at calf level
11. Change in bone metabolism [Time Frame: At baseline, during and after 5 days of dry-immersion]
   - Bone metabolism in response to immobilization by dry immersion assessed by measuring bone biomarkers in blood samples.

12. Change in Resting Metabolic Rate (RMR) [Time Frame: At baseline, during and after 5 days of dry-immersion]
- RMR measured by indirect calorimetry technique
13. Change in nitrogen balance [Time Frame: At baseline, during and after 5 days of dry-immersion]
- Nitrogen balance is a measure of nitrogen input minus nitrogen output. Nitrogen intake is calculated with a nutrition software. Protein oxidation measured in the 24-Hour urine collection estimates nitrogen output.
14. Change in Body Composition measured by DEXA [Time Frame: At baseline and at the end of the 5 days of dry-immersion]
- DEXA is a standard clinical technique to assess body composition
15. Change in glucose tolerance (Oral Glucose Tolerance Test) [Time Frame: At baseline and after 3 days of dry-immersion (to be comparable to a previous study on men)]
- Glucose and insulin levels measured at baseline (fasting) and 30, 60, 90, and 120 minutes after drinking within 5 min a water solution containing 75 g of glucose.
16. Change in Core temperature [Time Frame: At baseline and during the 5 days of dry immersion]
- Measured by electronic ingestible temperature capsules (e-Celsius Performance)
17. Change in psychological affects [Time Frame: At baseline, during and after 5 days of dry-immersion]
- Questionnaire used to assess the intensity of positive and negative affective states
18. Change in psychological state: sleep quality [Time Frame: At baseline, during and after 5 days of dry-immersion]
- Pittsburgh Sleep Dairy will be used to assess sleep perceived quality
19. Change in optic nerve sheath diameter (ONSD) considered as an indirect marker for intracranial pressure (ICP) estimation. [Time Frame: At baseline, during and after 5 days of dry-immersion]
- The optic nerve sheath diameter (ONSD) variations will be measured by echography.
20. Change in walking balance [Time Frame: At baseline and after five days of dry-immersion]
- Walking balance will be assessed by Dynamic Gait Index, specific parameter is: total Score (range 0-24). Higher scores mean a better outcome.
21. Change in standing balance [Time Frame: At baseline and after five days of dry-immersion]
- Standing balance will be assessed by posturography eyes open and eyes closed on a platform covered with 12-cm thick medium density foam.
22. Change in motion sickness susceptibility [Time Frame: At baseline and after five days of dry-immersion]
- Motion Sickness Questionnaire
23. Change in coagulation cascade [Time Frame: At baseline, during and after 5 days of dry immersion]
- Coagulation cascade in response to immobilization by dry immersion will be assessed by measuring coagulation parameters in blood.
